# Supplementary material for: Transcription dynamically patterns the meiotic chromosome-axis interface
Source: eLife. 2015 Aug 10;4:e07424. doi: 10.7554/eLife.07424 (PMC4530585; doi:10.7554/eLife.07424)
Supplement: Supplementary file 1. — Strains and primers. (A) Strains used in the work. (B) Primers used for qPCR and RT-qPCR. DOI: http://dx.doi.org/10.7554/eLife.07424.016 [file elife07424s001.docx]

**Supplementary File 1. Strains and primers.**

**A. Strains used in the work.**

| Strain Number | Genotype | Figure |
| --- | --- | --- |
| H119 | *MATa, ho::LYS2, lys2, ura3, leu2::hisG, his4B::LEU2, arg4-Bgl II*  *MATalpha, ho::LYS2, lys2, ura3, leu2::hisG, his4X::LEU2 (Bam)-URA3, arg4-Nsp* | *1,2,3,4,7,S1,S2,S3* |
| H6200 | *MATa, ho::LYS2, lys2, URA3, TRP, leu2::hisG, his3::hisG, REC114-13MYC::HIS3, rec8::HIS3MX6*  *MATalpha, ho::LYS2, TRP, LEU2, ura3, his3::hisG, REC114-13MYC::HIS3, rec8::HIS3MX6* | *4,7,S3* |
| H4471 | *MATa, ho::LYS2, lys2, ura3, leu2::hisG, TRP, his4B::LEU2, arg4-Bgl II, REC8::HA3::URA3*  *MATalpha, ho::LYS2, lys2, ura3, leu2::hisG, TRP, his4B::LEU2, arg4-Bgl II, REC8::HA3::URA3* | *2,S1,S5* |
| H6408 | *MATa, ho::LYS2, lys2, his3::hisG, URA3, leu2::hisG, TRP1, SMC4-Pk9::HIS3*  *MATalpha, ho::LYS2, lys2, his3::hisG, ura3, LEU, trp1::hisG, SMC4-Pk9::HIS3* | *S1* |
| H6309 | *MATa, ho::LYS2, lys2, his3::hisG, URA3, LEU2, TRP1, SMC3-3HA::HIS3*  *MATalpha, ho::LYS2, lys2, his3::hisG, ura3, leu2::hisG, TRP1, SMC3-3HA::HIS3* | *2,S1* |
| H7709 | *MATa, MATa, ho::LYS2, lys2, ura3, leu2::hisG, TRP, his4B::LEU2, arg4-Bgl II, YKL077W-URA3-YKL075C*  *MATalpha, ho::LYS2, lys2, ura3, leu2::hisG, TRP, his4X::LEU2-(Bam)-URA3, arg4-Nsp, YKL077W-URA3-YKL075C* | *1* |
| H7867 | *MATa, ho::LYS2, lys2, ura3::hisG, leu2::hisG, TRP, his4B::LEU2, arg4-Bgl II, YKL075C-URA3-YKL077W*  *MATalpha, ho::LYS2, lys2, ura3, leu2::hisG, TRP,*  *his4X::LEU2-(Bam)-URA3, arg4-Nsp, YKL075C-URA3-YKL077W* | *1* |
| H7907 | *MATa, ho::LYS2, lys2, ura3, leu2::hisG, TRP, his4B::LEU2, arg4-Bgl II, pCUP1-GAL2::KanMX6*  *MATalpha, ho::LYS2, lys2, ura3, leu2::hisG, trp, his4X::LEU2-(Bam)-URA3, arg4-Nsp, pCUP1-GAL2::KanMX6* | *S2* |
| H7908 | *MATa, ho::LYS2, lys2, ura3, leu2::hisG, TRP, his4B::LEU2, arg4-Bgl II, HIS3, pCUP1-GCY1::KanMX6*  *MATalpha, ho::LYS2, lys2, ura3, leu2::hisG, TRP, his4X::LEU2-(Bam)-URA3, arg4-Nsp, his3, pCUP1-GCY1::KanMX6* | *3* |
| H118 | *MATa, ho::LYS2, lys2, leu2::hisG, his4X::LEU2-URA3, ura3, arg4-nsp, dmc1Δ::ARG4*  *MATalpha, ho::LYS2, lys2, leu2::hisG, his4B::LEU2, ura3, arg4-Bgl2, dmc1Δ::ARG4* | *7* |
| H5494 | *MATa, ho::LYS2, lys2, leu2::hisG, his4X::LEU2-URA3, ura3, arg4-nsp, dmc1Δ::ARG4, pREC8::pREC8-SCC1-3HA::LEU2::rec8Δ::KanMX*  *MATalpha, ho::LYS2, lys2, leu2::hisG, his4B::LEU2, ura3, ARG4, dmc1Δ::ARG4, pREC8::pREC8-SCC1-3HA::LEU2::rec8Δ::KanMX* | *7* |
| H4590 | *MATa, ho::LYS2, lys2, leu2::hisG, ura3, ARG4, TRP1, HIS3(?), his4B::LEU2, dmc1Δ::ARG4, rec8::HIS3MX6*  *MATalpha, ho::LYS2, lys2, leu2::hisG, ura3, arg4-Bgl2, TRP1, HIS3(?), his4X::LEU2-URA3, dmc1Δ::ARG4, rec8::HIS3MX6* | *7* |
| H8038 | *MATa, ho::LYS2, lys2, URA3, leu2::hisG, his3::hisG, TRP1, pREC8::pREC8-SCC1-3HA::LEU2::rec8Δ::KanMX*  *MATalpha, ho::LYS2, lys2, URA3, leu2::hisG, HIS3, trp1::hisG, pREC8::pREC8-SCC1-3HA::LEU2::rec8Δ::KanMX* | *S5* |
| FK3870 | *MATa, ho::hisG, lys2, leu2::hisG, ura3, V5-RED1::hphMX4*  *MATalpha, ho::hisG, lys2, leu2::hisG, ura3, V5-RED1::hphMX4* | *5, S4* |
| FK4833 | *MATa, ho::hisG, lys2, leu2::hisG, arg4-Bgl2, ura3, hop1::LEU2 V5-RED1::hphMX4*  *MATalpha, ho::LYS2, lys2, leu2::hisG, ura3 , trp1::hisG, hop1::LEU2 V5-RED1::hphMX4* | *5, S4* |
| FK4815 | *MATa, ho::hisG, lys2, leu2::hisG, arg4-Bgl2, ura3, rec8∆::kanMX4, hop1::LEU2, V5-RED1::hphMX4*  *MATalpha, ho::hisG, lys2, leu2::hisG, arg4-Bgl2, ura3, rec8∆::kanMX4, hop1::LEU2, V5-RED1::hphMX4* | *5, S4* |
| FK4503 | *MATa, ho::LYS2, lys2?, ura3, leu2::hisG, red1::LEU2*  *MATalpha, ho::LYS2, ura3, leu2::hisG, trp1, his3, red1::LEU2* | *5, S4* |
| FK21 | *MATa, ho::LYS2, lys2, leu2::hisG, his4X, ura3, hop1::LEU2*  *MATalpha, ho::LYS2, lys2, leu2::hisG, his4B, ura3, hop1::LEU2* | *5, S4* |
| FK1091 | *MATa, ho::LYS2, lys2, ura3, leu2::hisG trp1::hisG REC8-HA3::URA3*  *MATalpha, ho::LYS2, his4, lys2, ura3, leu2::hisG trp1::hisG REC8-HA3::URA3* | *5, S4* |
| FK4396 | *MATa, ho::LYS2 lys2, ura3, leu2::hisG, trp1::hisG, red1::LEU, REC8-HA3::URA3*  *MATalpha, ho::LYS2 lys2, ura3, leu2::hisG, trp1::hisG, red1::LEU, REC8-HA3::URA3* | *5, S4* |
| FK5335 | *MATa, ho::LYS2, lys2, ura3, leu2::hisG, trp1::hisG, his3::hisG,*  *Red1-HA2-H3::KanMX, Rec8-HKMT::URA3*  *MATalpha, ho::LYS2, lys2, ura3, leu2::hisG, trp1::hisG, his3::hisG,*  *Red1-HA2-H3::KanMX, Rec8-HKMT::URA3* | *6* |
| FK5388 | *MATa, ho::LYS2, lys2, ura3, leu2::hisG, trp1::hisG, his3::hisG, Red1-HA2-H3::KanMX4, Rec8-HKMT::URA3 hop1::LEU2, pch2∆::NatMX4*  *MATalpha, ho::LYS2, lys2, ura3, leu2::hisG, trp1::hisG, his3::hisG,*  *Red1-HA2-H3::KanMX4, Rec8-HKMT::URA3 hop1::LEU2, pch2∆::NatMX4* | *S4* |
| FK5389 | *MATa, ho::LYS2, lys2, ura3, leu2::hisG, trp1::hisG, his3::hisG,*  *Red1-HA2-H3::KanMX4, Rec8-HKMT::URA3 hop1::LEU2*  *MATalpha, ho::LYS2, lys2, ura3, leu2::hisG, his3::hisG,*  *Red1-HA2-H3::KanMX4, Rec8-HKMT::URA3 hop1::LEU2* | *6* |
| FK5390 | *MATa, ho::LYS2, lys2, ura3, leu2::hisG, trp1::hisG, his3::hisG, Red1-HA2-H3::KanMX4, Rec8-HKMT::URA3, pch2∆::NatMX4*  *MATalpha, ho::LYS2, lys2, ura3, leu2::hisG, his3::hisG,Red1-HA2-H3::KanMX4, Rec8-HKMT::URA3, pch2∆::NatMX4* | *S4* |
| FK5332 | *MATa, ho::LYS2, lys2, ura3, leu2::hisG, trp1::hisG, his3::hisG,*  *Hop1-HA2-H3::KanMX, Rec8-HKMT::URA3*  *MATalpha, ho::LYS2, lys2, ura3, leu2::hisG, trp1::hisG, his3::hisG,*  *Hop1-HA2-H3::KanMX, Rec8-HKMT::URA3* | *6* |
| FK5391 | *MATa, ho::LYS2, lys2, leu2::hisG, ura3 , trp1::hisG, Hop1-HA2-H3::KanMX4, Rec8-HKMT::URA3, red1::LEU2, pch2∆::NatMX4*  *MATalpha, ho::LYS2, lys2, ura3, leu2::hisG, trp1::hisG, his3::hisG,*  *Hop1-HA2-H3::KanMX4, Rec8-HKMT::URA3, red1::LEU2, pch2∆::NatMX4* | *6* |
| FK5403 | *MATa, ho::LYS2, lys2, leu2::hisG, ura3 , trp1::hisG, Hop1-HA2-H3::KanMX4, Rec8-HKMT::URA3, pch2∆::NatMX4*  *MATalpha, ho::LYS2, lys2, leu2::hisG, ura3 , trp1::hisG, Hop1-HA2-H3::KanMX4, Rec8-HKMT::URA3, pch2∆::NatMX4* | *6* |
| FK5405 | *MATa, ho::LYS2, lys2, ura3, leu2::hisG, trp1::hisG, his3::hisG, Hop1-HA2-H3::KanMX4, Rec8-HKMT::URA3, red1::LEU2*  *MATalpha, ho::LYS2, lys2, ura3, leu2::hisG, trp1::hisG, his3::hisG,*  *Hop1-HA2-H3::KanMX4, Rec8-HKMT::URA3, red1::LEU2* | *6* |
| FK4393 | *MATa, lys2, leu2::hisG, V5-RED1::hphMX4, ura3, REC8-HA3::URA3*  *MATalpha, lys2, leu2::hisG, V5-RED1::hphMX4, ura3, REC8-HA3::URA3* | *6* |
| FK5273 | *MATa, lys2, leu2::hisG, ura3, ho::LYS2, trp1::hisG, arg4-Bgl2, REC8-HA3::URA3, hop1∆::TRP1, V5-RED1::hphMX4*  *MATalpha, lys2, leu2::hisG, ura3, ho::LYS2, trp1::hisG, arg4-Bgl2,*  *REC8-HA3::URA3, hop1∆::TRP1, V5-RED1::hphMX4* | *6* |
| FK5677 | *MATa ho::LYS2, lys2, leu2::hisG, his4, trp1::hisG, ura3, arg4-Nsp, REC8-HA3::URA3*  *MATalpha, ho::LYS2 lys2, leu2::hisG, his4, trp1::hisG, ura3, REC8-HA3::URA3* | *6* |
| FK1889 | *MATa ho::LYS2, lys2, leu2::hisG, ura3, trp1::hisG*  *prREC8-SCC1-HA3::LEU2::rec8∆::KanMX4*  *MATalpha ho::LYS2, lys2, leu2::hisG, ura3, trp1::hisG*  *prREC8-SCC1-HA3::LEU2::rec8∆::KanMX4* | *6* |
| FK5297 | *MATa, ho::hisG, lys2, leu2::hisG, arg4-Bgl2, ura3, V5-RED1::hphMX4, pREC8-SCC1-HA3::LEU2::rec8∆::KanMX4*  *MATalpha ho::LYS2, lys2,  ura3, trp1::hisG, V5-RED1::hphMX4, pREC8-SCC1-HA3::LEU2::rec8∆::KanMX4* | *6* |

**B. Primers used for qPCR and RT-qPCR**

| **Interval** | **Forward/**  **reverse** | **Sequence** |
| --- | --- | --- |
|  | | |
| **For Red signal at *URA3* insertions** (Fig. 1C) | | |
| Internal control | Forward | CTCGTGGAAAGATAACTAAACGGG |
|  | Reverse | GCATAAGGACAAACGACGCTC |
| **For Red signal without *URA3* insertions** (Fig. 1C left panel) | | |
| Interval 1 | Forward | CGAATGGATTGATAATGACAACGG |
|  | Reverse | TTGCTCCTTAGTAGGCTTAAACAC |
| Interval 2 | Forward | AGTGTTTAAGCCTACTAAGGAGCA |
|  | Reverse | GATGGCAATTCGGTGTACTTGTG |
| Interval 3 | Forward | AGCTTTAAACAAGAAACGCC |
|  | Reverse | GGTGAATGCATACTTCGAAGG |
| Interval 4 | Forward | CAC CCT TCC TCG AGA GGG CTG |
|  | Reverse | GGT ATC AAA TTG TCG TAC AGA TG |
| Interval 5 | Forward | AATAGAGAATGCATCAGCATCC |
|  | Reverse | ATTTAGCAGGAATGTGGAAGG |
| Interval 6 | Forward | CGCTCAACCACATTATCATCTCC |
|  | Reverse | ACCTGCCCTTGTAGATGTCT |
| Interval 7 | Forward | AGCAAACAAGACATCTACAAGGG |
|  | Reverse | TAGCCAAGAAAGACTGGAAAGAG |
| **For Red signal with forward *URA3* insertions** (Fig. 1C middle panel) | | |
| Interval 1 | Forward | CGAATGGATTGATAATGACAACGG |
|  | Reverse | TTGCTCCTTAGTAGGCTTAAACAC |
| Interval 2 | Forward | AGTGTTTAAGCCTACTAAGGAGCA |
|  | Reverse | GATGGCAATTCGGTGTACTTGTG |
| Interval 3 | Forward | AGCTTTAAACAAGAAACGCC |
|  | Reverse | GGTGAATGCATACTTCGAAGG |
| Interval 4 | Forward | CACCAACCAAGAGCCAAGAG |
|  | Reverse | CAGTGCTAACAACTTCATCAACAG |
| Interval 5 | Forward | CATTGTTGGTAGAGGATTGTTTGG |
|  | Reverse | CGCACTTAACTTCGCATCTG |
| Interval 6 | Forward | AATAGAGAATGCATCAGCATCC |
|  | Reverse | ATTTAGCAGGAATGTGGAAGG |
| Interval 7 | Forward | CGCTCAACCACATTATCATCTCC |
|  | Reverse | ACCTGCCCTTGTAGATGTCT |
| Interval 8 | Forward | AGCAAACAAGACATCTACAAGGG |
|  | Reverse | TAGCCAAGAAAGACTGGAAAGAG |
| **For Red signal with reverse *URA3* insertions** (Fig. 1C right panel) | | |
| Interval 1 | Forward | CGAATGGATTGATAATGACAACGG |
|  | Reverse | TTGCTCCTTAGTAGGCTTAAACAC |
| Interval 2 | Forward | AGTGTTTAAGCCTACTAAGGAGCA |
|  | Reverse | GATGGCAATTCGGTGTACTTGTG |
| Interval 3 | Forward | AGCTTTAAACAAGAAACGCC |
|  | Reverse | GGTGAATGCATACTTCGAAGG |
| Interval 4 | Forward | CATTGTTGGTAGAGGATTGTTTGG |
|  | Reverse | CGCACTTAACTTCGCATCTG |
| Interval 5 | Forward | CACCAACCAAGAGCCAAGAG |
|  | Reverse | CAGTGCTAACAACTTCATCAACAG |
| Interval 6 | Forward | AATAGAGAATGCATCAGCATCC |
|  | Reverse | ATTTAGCAGGAATGTGGAAGG |
| Interval 7 | Forward | CGCTCAACCACATTATCATCTCC |
|  | Reverse | ACCTGCCCTTGTAGATGTCT |
| Interval 8 | Forward | AGCAAACAAGACATCTACAAGGG |
|  | Reverse | TAGCCAAGAAAGACTGGAAAGAG |
|  | | |
| **For Red signal at *pCUP1-GAL2*** (Fig. S2B) | | |
| Interval 1 | Forward | TTTCATAATGGCAGTTGAGGAG |
|  | Reverse | TTCAGACATGGGCTTCTTGG |
| Interval 2 | Forward | GGCATCGCTGTCTTATGTCC |
|  | Reverse | CGTCTTCTACCTTATTCACCTCAC |
| Interval 3 | Forward | ATCTGCCATTAACTTCTACTACGG |
|  | Reverse | TGTCGTCATGTTGTAAATCCTC |
| Interval 4 | Forward | CCCTTATCAATGATATCCTTACG |
|  | Reverse | GATAAGTCTGGTGATGTGGTCC |
| Interval 5 | Forward | TCTTCCAGTTGAATTCCTGAG |
|  | Reverse | TTATCAGCCATTGATGAACCAG |
| Interval 6 | Forward | GCTCGAACCAAACTATCACTG |
|  | Reverse | TGGGTCACTCAAGGAAAGGA |
| Internal control | Forward | TTACTTGCTTACTTATCCACTCCC |
|  | Reverse | TGACATTCTGCGTTACAGTTTGG |
|  | | |
| **For Red signal at *pCUP1-GCY1*** (Fig. 3D) | | |
| Interval 1 | Forward | ACTCTTGTCAACAACCTTCC |
|  | Reverse | CGTGCTAGATTGATTCTACCC |
| Interval 2 | Forward | GCTACCGACACATTGATACTG |
|  | Reverse | CGTAGTCCAATCCTAACCTC |
| Interval 3 | Forward | GTGGTTGAAGCTTATTCTCCGT |
|  | Reverse | TTTGATTCGATCGGGATTCACAG |
| Interval 4 | Forward | GCGTGTTTCTCGTATGATTG |
|  | Reverse | GCAGGTAAAGTTTTCTTGCC |
| Interval 5 | Forward | ACGGTTTGCTTAGTTCTTACAC |
|  | Reverse | GTTTGGGCTACTTCTGGTGG |
| Interval 6 | Forward | CCTGCTCTCGAGTAGATGAC |
|  | Reverse | CGCGTAATTTCGAAGATTAACC |
| Internal control | Forward | TTAAGAAGGGAATGAGAAAGGG |
|  | Reverse | GAAGACGAGGATTAGAGGAGAC |
|  | | |
| **For RT-qPCR** (Figs. 3C, S2A) | | |
| *GAL1* | Forward | TAACAATGGCGGTATGGATCAG |
|  | Reverse | AGCTGTAGTGACTTCTACCA |
| *GAL2* | Forward | GGCATCGCTGTCTTATGTCC |
|  | Reverse | CGTCTTCTACCTTATTCACCTCAC |
| *SRL2* | Forward | GCTCGAACCAAACTATCACTG |
|  | Reverse | TGGGTCACTCAAGGAAAGGA |
| *GCY1* | Forward | GCTACCGACACATTGATACTG |
|  | Reverse | CGTAGTCCAATCCTAACCTC |
| *PFY1* | Forward | CCTGCTCTCGAGTAGATGAC |
|  | Reverse | CGCGTAATTTCGAAGATTAACC |
|  | | |
| **For Red1 signals on large/small chromosomes** (Fig. 5E) | | |
| *Position a - Chr3 219k* | Forward | TGGATGGCAACTGAAGGAGC |
|  | Reverse | TGGAATACCTATGAGTTGACTGC |
| *Position b - Chr1 171k* | Forward | CACTTCCTCGTGACCGTCTA |
|  | Reverse | ATTGCCAACTTTGACCACAA |
| *Position c - Chr1 195k* | Forward | GCATCAATGTACGCATCTACC |
|  | Reverse | GGCGATTCTGTCCAAGTAGC |
| *Position d - Chr6 216k* | Forward | TCGTATCAAAGAGAATTTGGATGA |
|  | Reverse | CGACTTTGATGCCTCTTTCC |
| *Position e - Chr1 95k* | Forward | TCGCCGAAGCATCTTGTAAT |
|  | Reverse | TGAGGCAAGTGCAAAGAATG |
| *Position f - Chr1 134k* | Forward | GCCATGAGAAGCAGAAGACA |
|  | Reverse | TGGCATACAATTCCAGTACTCC |
| *Position g - Chr1 156k* | Forward | GCGGTAAATTGTTGGGTGTT |
|  | Reverse | GCGGTTCAGCATCTCTTGAT |
| *Position h - Chr5 274k* | Forward | TTGACTGCTAACTGAGTTTTCATTTT |
|  | Reverse | TCGAATTTTTCTTTCCGTCA |
| *Position i - Chr4 435k* | Forward | ACATAGCGCGTCTACGGATG |
|  | Reverse | CACAAGCGGCAGGTATAGTG |
| *Position j - Chr4 712k* | Forward | GAAGCTTATGAACAAGAAGACCTC |
|  | Reverse | GGGATGCCTTGGGAACTATAA |
| *Position k - Chr7 506k* | Forward | CTACGAAGGAACCAGCTTGC |
|  | Reverse | CGAATGTGCACTACGAGCAT |
| *Position l - Chr16 576k* | Forward | TGGCAAAAAGAATCCGAAAA |
|  | Reverse | CGTCCCTCTGACGTTTTCTT |
|  | | |
| **For Red1-HA-H3 interaction with chromatin** (Fig. 6D) | | |
| *Position 1 - Chr3 219k* | Forward | TGGATGGCAACTGAAGGAGC |
|  | Reverse | TGGAATACCTATGAGTTGACTGC |
| *Position 2* -*Chr1 96k* | Forward | TCGCCGAAGCATCTTGTAAT |
|  | Reverse | TGAGGCAAGTGCAAAGAATG |
| *Position 3* -*chr1 134k* | Forward | GCCATGAGAAGCAGAAGACA |
|  | Reverse | TGGCATACAATTCCAGTACTCC |
| *Position 4* -*chr1 147k* | Forward | AAAATTTCTGAGTGAAGGGTGGT |
|  | Reverse | CATATCTTGGTGGCGCTGTA |
| *Position 5 -chr1 156k* | Forward | GCGGTAAATTGTTGGGTGTT |
|  | Reverse | GCGGTTCAGCATCTCTTGAT |
| *Position 6* *-chr3 55k* | Forward | TCGGTATTGTCACATCGTCAA |
|  | Reverse | TCAAGTGCCCTCATTGTTCA |
| *Position 7* *-chr3 93k* | Forward | TCCACGTAGGCGAAAGAAAC |
|  | Reverse | TTGACAACGACCAAGCTCAC |
|  | | |
| **For V5-Red1, Hop1 and Red1 localization** (Fig. S4) | | |
| *ADP1* | Forward | GGTGATGATTGCTCTCTGCC |
|  | Reverse | CGTCACAATTGATCCCTCCC |
| *Chr1 156k* | Forward | GCGGTAAATTGTTGGGTGTT |
|  | Reverse | GCGGTTCAGCATCTCTTGAT |
| *Chr5 274k* | Forward | TTGACTGCTAACTGAGTTTTCATTTT |
|  | Reverse | TCGAATTTTTCTTTCCGTCA |
| *Chr3 219k* | Forward | TGGATGGCAACTGAAGGAGC |
|  | Reverse | TGGAATACCTATGAGTTGACTGC |
