# Supplementary material for: Transcription dynamically patterns the meiotic chromosome-axis interface
Source: eLife. 2015 Aug 10;4:e07424. doi: 10.7554/eLife.07424 (PMC4530585; doi:10.7554/eLife.07424)
Supplement: Supplementary file 2. — Python code for sequence extraction for motif analysis. DOI: http://dx.doi.org/10.7554/eLife.07424.017 [file elife07424s002.docx]

**Supplementary File 2. Python code for sequence extraction for motif analysis.**

"""

Sequence Extraction for Motif Analysis

By Xiaoji Sun

This script extracts sequences from peak files to put into a fasta file and also generates a background fasta file containing ramdonly selected sequences from the whole genome.

This script accepts four inputs from commandlines and outputs a fasta file of

extracted sequences:

-g: sk1 genome sequence filename

-p: peaks.xls filename (from MACS)

-r: range of the region

-o: output fasta filename

-b: output filename of background sequences in fasta format

This scripts runs by the commandline:

e.g. python Sequence_extraction.py -g SK1_MvO_V1___GENOME/sk1_MvO_V1.fasta -p AH6407B_P15_peaks.xls -r 50 -o motif.fasta -b bg.fasta

"""

##################################################################################

# Modules

from Bio import SeqIO

import random

import optparse

##################################################################################

# Functions

def sequence_extraction(sk1_filename, peaks_filename, range_input, output_filename, bg_filename):

# reads the sk1 fasta file

sk1 = list(SeqIO.parse(sk1_filename, 'fasta'))

# reads the peaks file

f = open(peaks_filename, 'r')

# xls file

peaks = f.readlines()[24:]

f.close()

# puts the peaks data into a list of strings

for i in range(len(peaks)):

peaks[i] = peaks[i].strip().split('\t')

# calculates the summits positions and extract corresponding sequences

# summits in peaks xls file is off by two comparing to summits bed file

for i in range(len(peaks)):

for j in range(len(sk1)):

if peaks[i][0]==sk1[j].id:

peaks[i].append(str(sk1[j].seq[int(peaks[i][1]) + int(peaks[i][4])\

-2-range_input-1 : int(peaks[i][1]) + int(peaks[i][4]) -2 + range_input]))

# removes telomere regions (10kb)

peaks_notelo = []

for i in range(len(peaks)):

for j in range(len(sk1)):

if peaks[i][0]==sk1[j].name and (int(peaks[i][1])+int(peaks[i][4]))> 10000 and (int(peaks[i][1])+int(peaks[i][4])) < (len(sk1[j].seq)-10000):

peaks_notelo.append(peaks[i])

# sort the peaks by significance

#index = range(len(peaks_notelo))

#pvalues = [float(i[6]) for i in peaks_notelo]

#z = zip(pvalues,index)

#z.sort(reverse=True)

#index_sig = [i[1] for i in z]

# sort the peaks by fold-enrichment

index = range(len(peaks_notelo))

fe = [float(i[7]) for i in peaks_notelo]

z = zip(fe, index)

z.sort(reverse=True)

index_sig = [i[1] for i in z]

# outputs the sequences in a fasta format

output_lines = []

for i in index_sig:

output_lines.append('>'+'peak'+str(i+1)+'-'+peaks_notelo[i][0]+':'+\

str(int(peaks_notelo[i][1])+int(peaks_notelo[i][4])-2)+'-'+peaks_notelo[i][7]+'\n'+peaks_notelo[i][9])

f=open(output_filename,'w')

f.write('\n'.join(i for i in output_lines))

f.close()

# generates random sequences from the genome

bg_sequences = []

for i in range(5*len(peaks_notelo)):

chr_number = random.randint(1,16)

start_number = random.randint(1,len(sk1[chr_number-1].seq)-(range_input*2))

bg_seq = sk1[chr_number-1].seq[start_number : start_number+(range_input*2)]

bg_sequences.append('>'+'bgseq'+str(i+1)+'\n'+str(bg_seq))

# writes out background sequences

f=open(bg_filename,'w')

f.write('\n'.join(i for i in bg_sequences))

f.close()

###############################################################################

# Main

# parse object for managing input options.

parser = optparse.OptionParser()

# essential data, defines commanline options

parser.add_option('-g', dest = 'sk1_filename', default = '', help = 'This input\

is the fasta file of the sk1 genome sequences')

parser.add_option('-p', dest = 'peaks_filename', default = '', help = 'This input\

is the peaks.xls filename')

parser.add_option('-r', dest = 'range_input', default = '50', help = 'This input \

is the range on either side of summit position, default is 50(bp)')

parser.add_option('-o', dest = 'output_filename', default = '', help = 'This input \

is the output fasta filename')

parser.add_option('-b', dest = 'bg_filename', default = '', help = 'This input is the output background filename')

# loads the inputs

(options, args) = parser.parse_args()

# reads the inputs from command lines

sk1_filename = options.sk1_filename

peaks_filename = options.peaks_filename

range_input = int(options.range_input)

output_filename = options.output_filename

bg_filename = options.bg_filename

# runs the function

sequence_extraction(sk1_filename, peaks_filename, range_input, output_filename, bg_filename)
